# Estimating the potential of wild foods for nutrition and food security planning in tropical areas: Experimentation with a method in Northwestern Colombia

**DOI:** 10.1007/s13280-021-01624-9

**Published:** 2021-09-17

**Authors:** Jeferson Asprilla-Perea, José M. Díaz-Puente, Susana Martín-Fernández

**Affiliations:** 1 Universidad Tecnológica del Chocó “Diego Luis Córdoba”, Cra. 22 # 18b-10 B/ Nicolás Medrano, Ciudadela Universitaria, Quibdó, Chocó Colombia; 2 Universidad Politécnica de Madrid, Escuela Técnica Superior de Ingeniería Agronómica, Alimentaria y de Biosistemas, Avda. Puerta de Hierro 2, 28040 Madrid, Spain; 3 Universidad Politécnica de Madrid, Escuela Técnica Superior de Ingeniería de Montes, Forestal y del Medio Natural, Ciudad Universitaria, Madrid, Spain

**Keywords:** Nutrition and food security, Traditional use of biodiversity, Tropical forest, Wild food

## Abstract

Wild foods contribute to the food security of multiple communities in tropical areas of Africa, Asia and Latin America. However, wild foods are not regularly considered in the planning of strategies for food and nutrition security mainly due to the lack of technical and/or scientific knowledge so that they can be considered suitable for human consumption. This paper proposes a multidisciplinary method that estimates the potential of wild foods as alternative resources when planning interventions in favour of food and nutrition security in tropical forest territories. When designing the method, four dimensions were identified in science, technology and innovation (STI) that define this potential as well as ten assessment criteria. The wild foods chosen for applying the method were *Alibertia patinoi* (a fruit commonly known as Borojó) and *Proechimys semispinosus* (Mouse of thorns), which are two of the main wild foods traditionally used by human communities in a tropical forest territory in the northwest of Colombia. In both cases, although there are significant advances in STI, compliance with some criteria is still required to regard them as viable alternatives for nutrition and food security within this territory. This research is useful for promoting the inclusion of wild food in food security programmes for communities where this food is already included in their traditional pattern of consumption and identifies the progress needed in STI to achieve this purpose. It may also promote the early recognition of possible traditional and cultural practices with high risk of transmission of pathogenic elements by the handling and/or inadequate consumption of wild foods. This early recognition could contribute to the prevention of diseases of wild animal origin, including those of rapid global spread.

## Introduction

Tropical forests provide many of the basic needs of nearly 800 million people that live in them (Groom and Palmer 2012; Kashwan and Holahan 2014), of which an estimated 38% are undernourished (FAO 2015). These forests are globally important for their high biodiversity and level of endemism (Malhi and Grace 2000; Groombridge and Jenkins 2002); the environmental services they provide, such as the capture and processing of significant amounts of carbon (Wright 2010); and the contributions to the diet of diverse communities through food obtained from domestic or wild species (Cruz et al. 2013, 2014; Álvarez-Salas 2014).

Wild foods are food products obtained from non-domesticated species. These products may be harvested (gathered or hunted) from within food and agricultural production systems or from other ecosystems (Heywood 1999; FAO 2019; Borelli et al. 2020). Within this particular group of foods are cereals, vegetables, fruits, tubers, eggs, meats and others (Misra et al. 2013; Schulp et al. 2014; Termote et al. 2014; Erskine et al. 2015; Fa et al. 2015; Acosta-Naranjo et al. 2020). The sowing or breeding of species under ancestral production schemes does not necessarily eliminate its wild condition. In order for a species to stop being wild, it must be verified that none of its populations exist in its natural state making it a strictly a domesticated species. Domestication species are understood to be species bred in captivity or in an “artificial” environment. These species are, therefore, modified from their wild ancestors so that they can be more useful or pleasant to human beings who control their key biological aspects—such as their reproduction and, in the case of animals, their diet (Diamond 2002).

In the context described above and from a biological perspective, domestication refers to a category of species and not to individuals. This aspect is of great importance when understanding the concept of wild foods. These foods can be reproduced, without changing their genetic configuration as a species, at the individual level through sewing (plants) or rearing (animals) in experimental management units or through traditional practices in different rural areas of the planet.

Different local studies associated with tropical forest areas, demonstrate the use of plants (Binu 2010; Narayanan et al. 2011; Pauro et al. 2011; Keatinge 2012; Martínez-Pérez et al. 2012; Chandra et al. 2013; Cruz et al. 2013; Grados and Peláez 2014; Saha et al. 2014; Bortolotto et al. 2015; Borelli et al. 2020; George and Christopher 2020) and wild animals (Robinson and Bennett 2000; Townsend and Rumiz 2003; Asprilla-Perea and Hinestroza 2011; Nasi et al. 2011; Asprilla-Perea et al. 2012; Kamga et al. 2013; Misra et al. 2013; Álvarez-Salas 2014; Cruz et al. 2014; Fa et al. 2015; Asprilla-Perea and Díaz-Puente 2019; FAO 2019) in feeding multiple communities in Africa, Asia, Latin America and the Caribbean.

On the other hand, wild foods are not regularly considered in the planning of strategies in favour of nutrition and food security (FAO 1996; Weingärtner 2005; Garnett 2014) due to insufficient knowledge of their sustainability and health questions regarding human consumption. To address the knowledge gap that limits the inclusion of wild foods in food and nutrition security planning, the academic community has been making multiple efforts. These efforts are recognized in various specific disciplinary studies that define ethnobiological aspects (Robinson and Bennett 2000; Townsend and Rumiz 2003; Van den Eynden al. 2003; Asprilla-Perea et al. 2012; Misra et al. 2013; Schulp et al. 2014; Termote et al. 2014; Erskine et al. 2015; Fa et al. 2015); evaluate the nutritional value of foods and possible transformation techniques in products with commercial potential (Leterme et al. 2006; Tejada et al. 2006; Bustacara and Joya 2007; Palomino et al. 2010; Torres-Rapelo et al. 2014; Uchôa-Thomaz et al. 2014; Álvarez-Salas 2014; Serpa al. 2015; Alvis et al. 2017; Phan et al. 2020); or describe and/or promote ancestral planting or breeding practices (Larrazábal 2004; Viloria and Córdova 2008; Suárez et al. 2009; Cifuentes et al. 2010; Álvarez et al. 2015; Sicchar-Valdez et al. 2015).

In this sense, some indices have been developed to know the cultural significance of wild foods such as the Smith relevance index based on free-list that measures botanical cultural knowledge (Smith 1993), cultural food significance indices (Pieroni 2001; Alonso-Aguilar et al. 2014) that evaluate availability, typology of the used parts, frequency of use, kind and number of food uses, taste appreciation, knowledge transmission and perceived role as a food-medicine from the local population point of view and the Food Significance Index and the Salience Index food importance index (Pío-León et al. 2017) which determine plant importance by the measure of culinary diversity to identify the priority species, all these indices are based on consumer information and preferences and are focussed on plant species. They do not consider nutrition and food security or scientific results in their formulation. Therefore, as in this study, we propose the inclusion of other evaluation factors such as the sustainability of the local harvesting model and the nutritional assessment of foodstuffs. Likewise, the proposed index offers a broader application option over the traditional use of biodiversity, since it not only allows its application to plants, but also to fungi and animals (vertebrates and invertebrates). This proposal is generally considered a contribution to improve the results of these tools to prioritize wild species with potential for safe food and nutrition.

The main background to this work is Asprilla-Perea et al. (2020), in which four dimensions in Science, Technology, and Innovation (STI), and ten criteria linked to them were identified to establish the potential of wild foods for responsible consumption. Continuing the approach of Asprilla-Perea et al. (2020), this work proposes and applies a multidisciplinary qualitative method, which integrates these dimensions and criteria, for estimating wild foods potential as alternatives to cultivated (commercial) foods when planning food and nutrition security in tropical forest territories where this food is already included in their traditional pattern of consumption.

## Materials and methods

### Study area

**Fig. 1:**
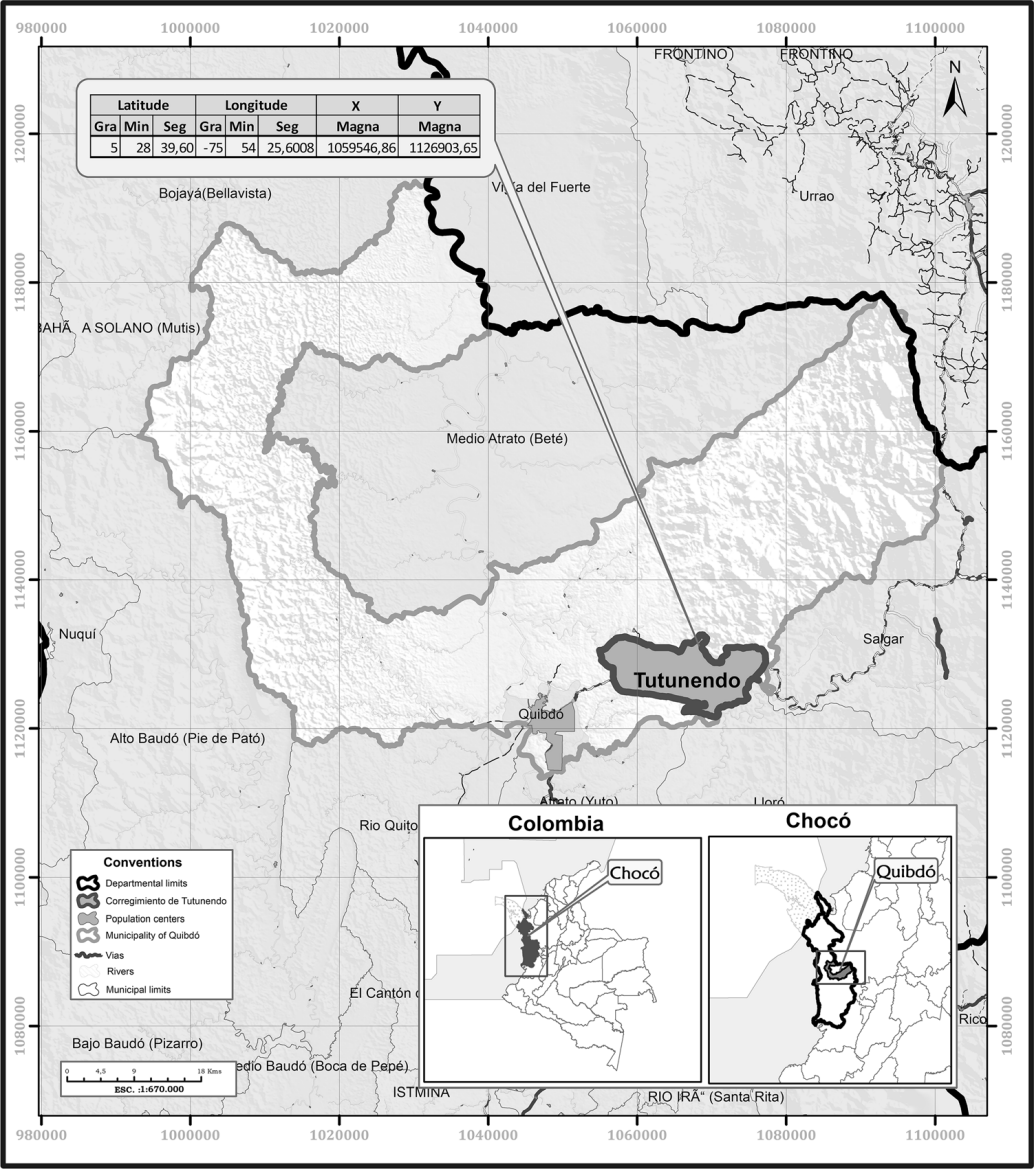
Geographical location of the study area in Northwestern Colombia

The experimentation of the method (within its development process) was carried out in the town of Tutunendo, which is located at 5°28′39″ N and 75°54′25″ W in the municipality of Quibdó, department of Chocó (Northwestern Colombia) (Fig. 1). This territory is located at 100 masl and has an extension of 43 km^2^.

The town has a population of approximately 3500 inhabitants, the majority being Afro-descendants (Cuesta-Ríos et al. 2007). Tutunendo belongs to the tropical rain forest ecosystem, physiographically located in the area called Central Rain Forest, in the Chocó Biogeographic. It has an average annual precipitation of 11,394 mm; average temperature between 27 and 30 °C; and a relative humidity of approximately 90%. According to Eslava (1994), this is one of the rainiest places in the world.

### Theoretical and practical design of the method

The experimental development process draws from the approach defined by Asprilla-Perea et al. (2020) for estimating the potential of wild foods from the scientific and technological dimensions of them. The method was applied experimentally in the town of Tutunendo, in the department of Chocó. This experimentation allowed the theoretical method to be calibrated to better reflect reality and show the practical application in a territory associated with tropical forests in Northwestern Colombia. In the following sections, the method application process and its respective experimentation are presented.

### Dimensions and criteria for evaluating the potential of wild foods

Four dimensions and ten evaluation criteria constitute the theoretical basis of the method (Asprilla-Perea et al. 2020). It consists in checking the compliance with the minimum conditions established in the ten evaluation criteria of the dimensions in STI that define wild foods potential when planning interventions in favour of food and nutrition security in tropical forest territories. One criterion is fulfilled if there are scientific works in the criterion area to back it up. Table 1 details these dimensions and their respective evaluation criteria.

**Table 1:** Dimensions and criteria for evaluating the potential of wild foods

Dimensions	Assessment criteria	Type of studies	Discipline
1. Importance of food for the community that consumes it	1.1 Food is traditionally consumed in the territory^a^	Ethnobiological study	Sociology Anthropology Biology Ecology
	1.2 The pattern of use of the wild food in the territory is known: forms of use (raw food or in some kind of preparation); parts or by-products used as food; description of culinary preparation (when applicable); frequency of family consumption in the territory, etc	Ethnobiological study	Sociology Anthropology Biology Ecology
	1.3 The consumption preference of this food has been technically demonstrated with respect to at least one non-wild food of the same food group (Pett 1942) within the family basket of the territory	Economic study. In this criterion, the community's behaviour in relation to the consumption of wild food should be evaluated in view of its possible inclusion in the planning of interventions in favour of the territory's nutrition and food security. The objective is to achieve a reasonable level of certainty and to find out to what extent wild food satisfies the needs of consumers with respect to other products within the same food group	Economics
2. Sustainability of the local use model of the wild food	2.1 The traditional sources for obtaining food for family consumption in the territory are known	Ethnobiological study or economic study	Sociology Anthropology Biology Ecology Economics
	2.2 There are proven mechanisms for sustainable use of the wild food in the territory. These mechanisms ensure the availability of the food without risking the conservation of the wild species from which it is obtained or for any other species that coexist in its environment. The mechanisms are in line with the applicable wildlife protection laws of the respective country^a^	Studies on forest management for harvesting purposes (sustainable harvest rates; traditional systems of production of plants, fungi or animals; experimental development of production processes of plants, fungi or animals; forest management)	Sociology Anthropology Forestry Ecology Agronomy Zootechnics and Veterinary Economics Natural resources law
	2.3 Positive cost/benefit analysis of the mechanisms for sustainable use of wild food tested in the territory	Economic study	Economics
3. Nutritional value and risks to human health	3.1 Wild food having a nutritional value with similar or higher quality (in at least one bromatological characteristic) with respect to a non-wild food of the same group. The results should be obtained through samples of the wild food obtained in the territory^a^	Bromatological studies (composition and nutritional properties; physical and chemical changes of the food)	Chemistry Physics Physiology Microbiology Biochemistry
	3.2 Studies in the territory about nutrient assimilation of wild foods demonstrated no assimilation problems (Saunders 1960)	Human nutrition studies	Nutrition
	3.3 Wild food with studies in the territory showing that its consumption does not entail risks to human health^a^	Bromatological studies (verification of hygiene and quality standards, revision of the legislation concerning quality control)	Chemistry Physics Physiology Microbiology Biochemistry
4. Processing techniques for products with commercial potential	4.1 Wild food with at least one experimental development and/or technological development (carried out in the territory) that allows its transformation into products with commercial potential	Experimental development and/or technological development of products with commercial potential from wild foods	Food Technology

*Maximum relevance criteria

In Table 1, we consider community consumption preference as the culturally transmitted nutritional believes, values, preferences and modes of preparation that make the people of the community to choose a wild food rather than a non-wild food of the same group (Rozin 1990). This is important because the traditional consumption of wild foods is often due to the greater availability in the territory with respect to other non-wild foods that must be entered from other sites. Recognizing these types of aspects would allow us to understand the behaviour of the community in case of having equal availability in the territory of non-wild foods from the same group to which the evaluated food belongs.

The proposed criteria attempt to evaluate different aspects of wild foods for possible inclusion in interventions for food security in the territory; its results allow a balance of aspects such as consumer preference, sustainability of availability, and nutritional quality between wild and non-wild foods from the same group. These are key aspects for the relevance and sustainability of policies, plans, programmes and projects to guarantee the food and nutritional security of territory. We also take into consideration the positive cost/benefit analysis of the mechanisms for sustainable use of the wild food. In case the cost of production/collection of the wild food (whatever the mechanism defined) is higher than the benefits in terms of access and/or availability of the product, this result would not be positive and would not comply with the criterion. Under this outcome, the wild food could contribute to the unsustainability of the intervention that includes it to try to improve food and nutrition security.

When interpreting the results of the method, criterion 1.1 (food is traditionally consumed in the territory) was established as mandatory, and the following three criteria were classified to be of maximum relevance: 2.2 there are proven mechanisms for sustainable use of the wild food in the territory; 3.1 wild food having a nutritional value with similar or higher quality (in at least one bromatological characteristic) with respect to a non-wild food of the same group; and 3.3 wild food with studies in the territory showing that its consumption does not entail risks to human health.

The selection of these four maximum relevance criteria as the fundamental basis for interpreting the results of the method is articulated in Asprilla-Perea and Díaz-Puente (2019). This research indicates that the main challenges in STI to consider wild foods as a viable alternative in food and nutrition security planning for tropical forest territories are related to species conservation and human health risks. The multicriteria method AHP (Analytic Hierarchy Process) applied to a group of experts in Asprilla-Perea et al. (2020) pointed to the following challenges: the importance of food for the community; the guarantee of a local model of sustainable use of food; and the evaluation of the nutritional value and risks to human health from the management and consumption of wild foods. These are essential variables for determining the responsible consumption of these types of foods with respect to human health and nature conservation.

### Method for evaluating the potential of wild foods

**Fig. 2:**
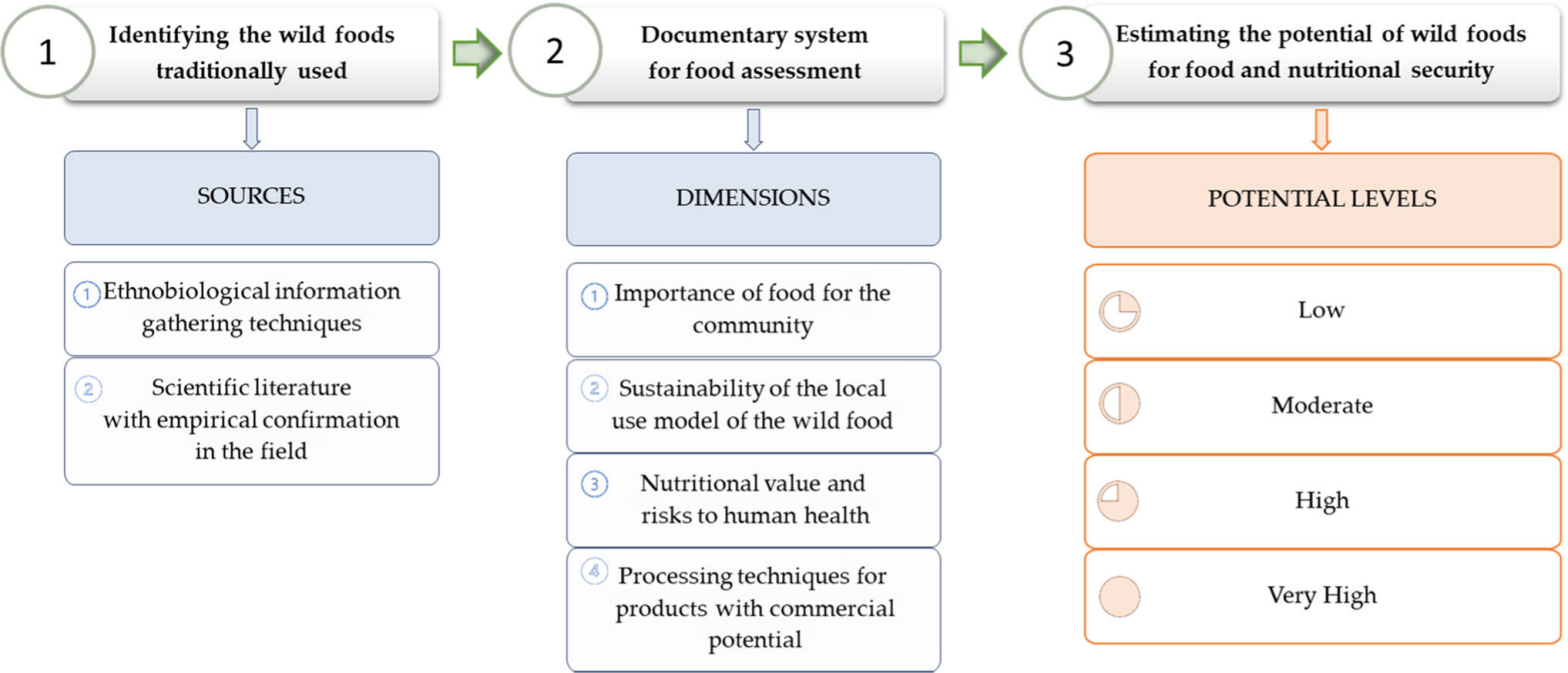
Method for estimating the potential of wild foods for nutrition and food security: steps for its implementation

The proposed method allows for the evaluation of the four dimensions in STI using the ten defined criteria. The application of this method is carried out by executing three steps (Fig. 2):

1. Identify the wild food traditionally used in the territory. This process can be carried out through ethnobiological information gathering techniques in the territory or by reviewing the scientific literature generated. In the second case, empirical confirmations must be made in the field before continuing with the other steps of the method.
2. Create a documentary system for food evaluation. This system can be built from primary information supplemented with secondary information or vice versa. However, because of time consumption and economic resources, the ideal would be the formation of large human teams with an interdisciplinary profile that allows the generation of primary information for the evaluation of the 10 criteria. These can then be supported and/or discussed with secondary information.
3. Apply the evaluation criteria to food. In this process, the advances in STI for the food being studied are described and compliance is determined with respect to each criterion.

We propose to classify the fulfilment of the evaluation criteria with four levels: Low Potential, Moderate Potential, High Potential or Very High Potential (Fig. 2, Table 2). This would be the main outcome of the method that can assist decision-making. The four levels allow an easy reading to identify the wild food strengths regarding its potential and the feasibility of including the wild food in the food security planning of the territory: (1) it could be feasible when the evaluation result is Very High Potential; (2) feasible but needs some previous research work when the result is High Potential; (3) not easily feasible and in need of much work when the result is Moderate Potential; and (4) disposable when the result is Low Potential. As a complementary outcome, the method allows the identification (with a territorial approach) of the needs in STI of the evaluated food. This could be considered a roadmap with research questions and technological and innovative challenges for the scientific community to address the elements that maintain the knowledge gap in a pertinent way and limit the inclusion of these wild foods in food and nutrition security planning. Thus, the estimation of the potential of a wild food through the proposed method consists of the integration of the main outcome and its complementary outcome. The main outcome provides an easy reading of the wild food feasibility to support the work of food policy planners; and the complementary outcome identifies the possible challenges that limit the inclusion of the wild food in the food policy planning (in the case that the main result is not Very High Potential).

**Table 2:** Interpretation model of the method results (MrC: Maximum relevance Criteria; Ac: Assessment criteria; VHP: Very High Potential; HP: High Potential; MP: Moderate Potential; LP: Low Potential)

Evaluation of the result	Description of the result	Summary
Very high potential	The wild food fulfils the ten criteria defined in the method, which allows it to be considered a viable alternative when planning nutrition and food security in tropical forest territories. In this sense, food is important for the community that consumes it; has a nutritional value similar to or better than that of at least one non-wild food of the same group; its intake does not entail risks to human health; appropriate techniques for its sustainable use are known; and progress is made in the development of products with commercial potential	4 MrC and 6 Ac → VHP
High potential	The Wild food is traditionally consumed in the territory, and according to the assessment criteria, although it does not yet meet all the minimum conditions of the ten criteria to be considered a viable alternative when planning nutrition and food security in the territory, it has important advances in STI that indicate it meets not only the maximum relevance criteria (1.1; 2.2; 3.1; 3.3), but at least one additional criterion	4 MrC and at least1 Ac → HP
Moderate potential	The wild food is traditionally consumed in the territory. It cannot yet be suggested as an alternative when planning nutrition and food security because it only meets the maximum relevance criteria	4 MrC and 0 Ac → MP
Low potential	The wild food is traditionally consumed in the territory but should not be suggested as an alternative when planning nutrition and food security because it does not meet all the maximum relevance criteria	Less than 4 MrC → LP
Important notes on the interpretation of the results of the implementation of the method designed		
1. The method is only applicable to the assessment of the potential of wild food in tropical forest territories		
2. The potential of a wild food may change upwards or vice versa according to advances in STI during the time between measurements		
3. Like any method, the reliability of the outcome of its application will depend on the veracity of the data included in its assessment		

### Experimental application of method

Experimentation of the method designed and proposed in this study was carried out by applying it to *Alibertia patinoi* (Cuatrec.) Delprete & C.H. Perss and *Proechimys semispinosus* (Tomes 1858), which, according to Asprilla-Perea and Díaz-Puente (2018) are two of the main wild foods used in the municipality of Tutunendo, located in the municipality of Quibdó, northwest of Colombia.

*Alibertia patinoi*, known locally as “Borojó”, is a plant-originated food that is obtained from a tree species of the Rubiaceae family, native to tropical areas of America. Its geographical distribution is limited to the centre of the Equator climatic zone. The fruit is a fleshy berry from 7 to 12 cm in diameter, which, in its first stages, is light green and when ripe is a reddish brown. It has a fleshy mesocarp with aromatic flavour and is highly perfumed (Giraldo et al. 2004; Díaz-Ocampo et al. 2012). The fruits have an average weight of 740 g and consist of seed, pulp and peel. Seeds often constitute up to 10% of the fruit weight (Jaramillo et al. 2005).

*Proechimys semispinosus*, known locally as “Mouse of thorns”, is a species of rodent typical to humid and dry tropical forests. On average, this mammal is born with an approximate weight of 23 g and can reach up to 500 g in its adult state. Its longevity in the natural state reaches up to 5.5 years. This species has a high reproductive rate because it has two to six pups per birth and pups can be born as many as four times a year. The feeding of this species is usually based on fruits and seeds (Elizondo 1999; Jiménez-Ortega et al. 2007).

Due to the multidisciplinary nature of the STI dimensions and the technical and/or scientific complexity required for their comprehensive assessment, the information regarding the minimum evaluation criteria for the two wild foods selected in this study was obtained through the gathering of primary information in the field on ethnobiological aspects and a systematic review of literature on socioeconomic, bromatological, human nutrition and food science and technology aspects.

For the field survey for ethnobiological information about wild foods, a questionnaire based on direct questions was prepared (Table 3). It was applied in the form of an interview to key informants of Tutunendo. Key informants were identified based on criteria of expertise in the area with the support of community leaders. The criteria taken into account for the selection of informants were (a) to have lived in the local area for more than 30 years and (b) to be recognized there as a “local knowledge holder”. Local knowledge holders are highly respected for their experience and knowledge in sociocultural aspects within black and indigenous communities in Northwestern Colombia. Among the local knowledge holders interviewed were farmers, hunters, housewives and traditional doctors; 11 were men 4 women and all of them older than 50 years of age. All identified informants were interviewed (15 in total).

**Table 3:** Questionnaire applied to key informants

Questions (Qni)	
Q1	What are the species of plants, fungi and animals of the forest that you know are consumed as food in your community?
Q2	How do you use the plant, fungus or animal in this community?
Q3	What is the part or by-product of the plant, fungus or animal that is used as food in this community?
Q4	From where the plant, fungus or animal is obtained for consumption in this community?
Q5	Do you know about the current or past existence of cultivation/farms of the plant, fungus or animal for consumption as family food in this community?
Q6	Do you know about the current or past existence of cultivation/farms of the plant, fungus or animal for sale as food in this community?
Q7	Do you know about the current or past existence of any study to help the cultivation/breeding of the plant, fungus or animal in this community?
Q8	Do you know about any measure of the environmental authority (CODECHOCO) to protect the species of plant, fungus or animal in this community?
Q9	In which season, is this food available for consumption in the community?
Q10	Do you know about the current or past existence of any disease or discomfort to human health due to the consumption of the plant, fungus or animal in this community?

This questionnaire was not elaborated and applied only for the two species that are included in the article. Ethnobiological information was obtained for all wild species recognized as food by key informants

The literature review allowed information to be obtained to evaluate the criteria that were not assessed through primary information in the field. Instead, these evaluations were carried out through accessing documents on the Web of Science and through other search engines including Google Scholar. Likewise, we reviewed the repositories of undergraduate and thesis work found in the Faculties of Natural Sciences and Engineering of the Technological University of Chocó, which is the main institution of higher education with research activities in the area. For inclusion of documents, the following criteria were used: (a) documents were published in peer-reviewed journals or graduation/thesis papers with empirical research results; (b) documents enabled the assessment of at least one of the minimum criteria associated with dimensions; and c) studies had been conducted in the village of Tutunendo.

With the information obtained, the assessment criteria were applied to *Alibertia patinoi* and *Proechimys semispinosus* as established by the method proposed in this study. During this process, some assessment criteria were technically adjusted in terms of their drafting and scope, which was necessary to improve their applicability and replicability capabilities**.**

## Results

### Application of the method to two wild foods in Northwestern Colombia

**Fig. 3:**
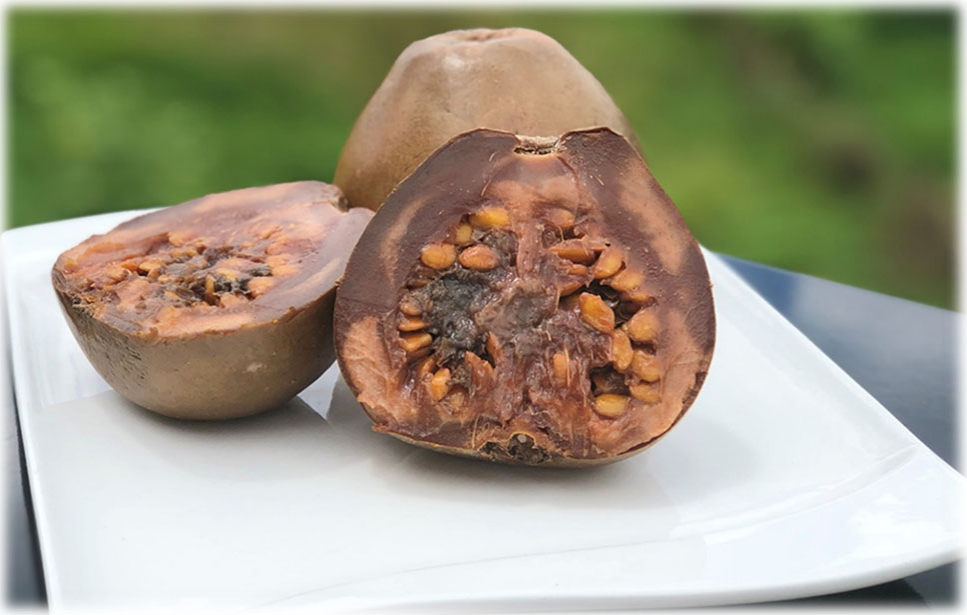
Fresh fruit of *Alibertia patinoi* from the rain forest in the Northwestern Colombia. Photo by Evelin Couttin

Although the fruit of *Alibertia patinoi* (Fig. 3) does not yet meet all positive results in the evaluation of criteria to be considered as a viable alternative when planning food and nutrition security for the village of Tutunendo (Northwestern Colombia), there are significant advances in STI that resulted in a High Potential after the implementation of the method. In this sense, the importance of Borojó is recognized as a food of the inhabitants in this territory. It has nutritional attributes similar to that of other non-wild fruits such as the apple, strawberry, pineapple or papaya. Its intake does not entail risks to human health. Appropriate techniques are known for sustainable use and progress is being made in the development of products with commercial potential. To meet all the minimum criteria, *Alibertia patinoi* requires studies with positive results on the preference of consumption with respect to other non-wild food of the same group in that territory and on the biological assimilation of this food in inhabitants of the area. In Table 4, the results obtained in the evaluation of the potential of *Alibertia patinoi* (Borojó) are presented.

**Table 4:**
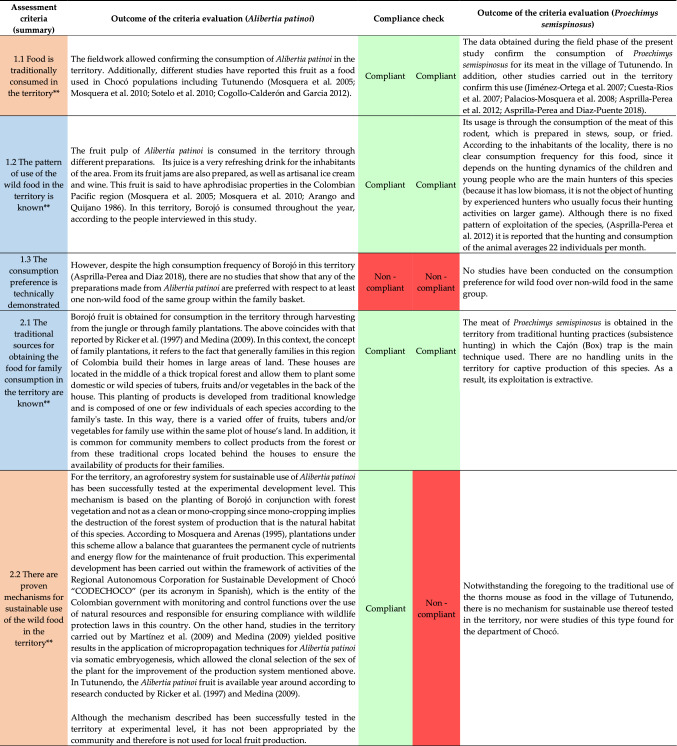

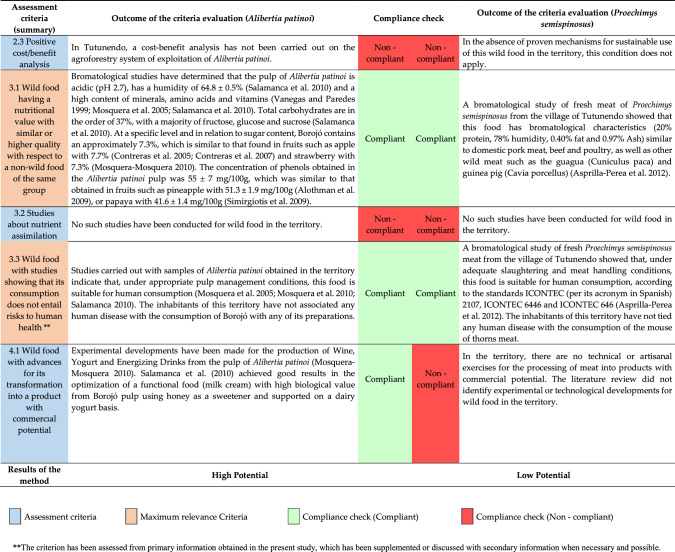
Results of the estimation of the potential of *Alibertia patinoi* and *Proechimys semispinosus* through the application of the method designed in Northwestern Colombia

**Fig. 4:**
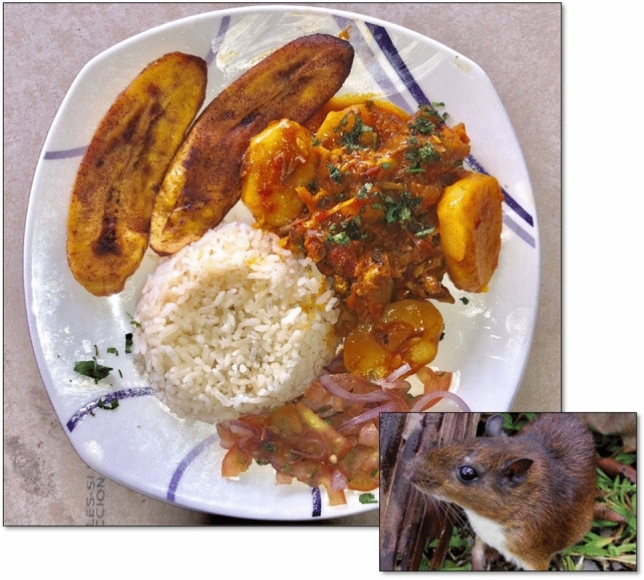
Dish prepared with meat from *Proechimys semispinosus* from the rain forest in Northwestern Colombia. Photos by Jeferson Asprilla (dish) y Alex M. Jiménez (animal)

In the case of *Proechimys semispinosus* (Fig. 4), there are some advances in STI to recognize its potential as an alternative when planning nutrition and food security for the village of Tutunendo (northwest of Colombia). The result indicates that this food has a Low Potential, which is mainly due to the lack of sustainable use mechanisms that guarantee its availability in the territory without jeopardizing the conservation of the species or any other species that coexist with it in its natural environment. It also lacks studies on consumption preference over other non-wild foods of the same group; positive results in biological assimilation studies; and advances in processing techniques in products with commercial potential. In Table 4, the results obtained in the assessment of *Proechimys semispinosus*'s potential (Spines Mouse) are presented.

## Discussion and conclusions

The method proposed in this study offers a new alternative in evaluating the potential of wild foods and is articulated with the approach of Pío-León et al. (2017) who suggest that ethnobiological indices that value the cultural importance of this type of food are not sufficient on their own to adequately define the potential of wild foods.

As shown in this study, the method designed is a viable method for evaluating the potential of wild food in tropical forest territories to which it assigns one of these four values: Low Potential, Moderate Potential, High Potential and Very High Potential for classifying wild food. These levels indicate the advances in STI on these foods that allow them to be considered viable alternatives in planning food and nutrition security with a rural/local focus, which is consistent with the Integrated Approach for Conserving and Sustainably Using WFPs proposed by Borelli et al. (2020), who indicate that wild foods evaluated multidisciplinary through the CTeI and properly managed, could be introduced in national food and nutrition security and sovereignty strategies that focus on nutrient adequacy rather than quantity of staples, while being culturally acceptable and relevant for each territory.

One arguable issue of the method is how to interpret the lack of information. A criteria for which there are no data is judged as non-compliant in the estimation of the potential of the wild food (see criteria 2.3 in the application to *Alibertia patinoi* and criteria 1.3 in the application to *Proechimys semispinosus*). This valuation could be interpreted as a bias towards low potential in the results when data are missing. However, it is important to understand that the method yields two outcomes: (1) the main outcome that provides an easy reading of the wild food strengths regarding its potential and its feasibility to be included in food policy planning; and (2) the complementary outcome that identifies possible challenges that limit this inclusion. The assessment of wild food as non-compliant in a particular criteria informs decision-makers about the unfeasibility of the wild food for food security planning, either because of its negative results with the existing data or due to the lack of data. In this way, the method highlights both, the negative results and the importance of this lack of information, something that the present research seeks to emphasize to encourage the progress needed in STI. In any case it is not final assessment about wild food potential. As Table 2 indicates, the potential of a wild food may change upwards or vice versa according to advances in STI during the time between measurements.

On the understanding that planning is a political/administrative decision-making exercise, it is preferable that the application of the method includes a focus on municipalities, communities or other territorial organization. However, when considering the scale of the secondary information source for the evaluation of the criteria, it is important to specify that in this study the territorial reference is based on an ecosystem classification and not on a political/administrative one. In this sense, an ecosystem is understood as a biological system constituted by a community of living organisms (biocenosis) and the physical environment where they are related (biotope) or as a unit composed of interdependent organisms that includes man and share the same habitat (Tansley 1935; Molles 1999; Smith and Smith 2012). From this perspective and considering the approach of Armenteras et al. (2016), ecosystems become important in regional decisions and management as long as they are identified and delimited in the territory and integrated with the other social, cultural and economic elements (Crespin and Simonetti, 2019). Data from secondary information obtained from other territories can always contribute to the discussion of the results, but it is preferable that the assessment of the criteria be based on locally obtained data.

As a complementary result—in the event that the potential of the food is not very high—the method designed allows the identification of the needs of the evaluated food in STI so that it is known whether it should be included or not in the diet of the rural population of the tropical forest areas. Therefore, using STI defines a roadmap with research questions and technological and innovative challenges for the scientific community to address in a relevant way.

The model for interpreting the results of the method proposed in this study (Low, Moderate, High and Very High Potential) is based on the relationship between the ten criteria that evaluate the four dimensions in STI. These dimensions define the potential of wild foods and the main concerns of the scientific community about the fact that these foods can be considered as a viable alternative when planning nutrition and food security of tropical forest territories where their consumption by cultural tradition is a reality. According to Asprilla-Perea and Díaz-Puente (2019), the main challenges or concerns are related to possible negative effects and risks.

The negative effects would be related to biodiversity conservation as a result of unplanned extraction practices. These practices may lead to excessive use of plants, fungi and/or wild animals that could cause local population reductions and/or habitat fragmentation in the medium and long term, directly or indirectly affecting ecosystem functioning (Robinson and Bennett 2000; Fa and Peres 2001,2001; Peres 2001; Bennett and Rao 2002; Fa et al. 2003, 2005; Gardner et al. 2006; Laurance et al. 2006; Wright and Muller-Landau 2006; Fa and Brown 2009; Ziegler 2010).

The possible risks would be related to human health due to the lack of assessment of the nutritional and health quality of wild foods. In this sense, the frequent consumption of food without recognition of its nutritional and health quality can not only generate health risks through the viral and bacterial disease transmission such as HIV, EBOLA or COVID-19 (Cawthorn et al. 2020; McNeely 2021), among others, but also through imbalances in human nutrition (Feng et al. 1999; Bell et al. 2004; Leroy et al. 2004; Karesh et al. 2005; Pandey et al. 2006; Asprilla-Perea et al. 2012; Keatinge 2012; Kamga et al. 2013).

Based on these elements and the conclusions of Asprilla-Perea et al. (2020), three criteria of maximum relevance and one mandatory criterion were selected from the ten criteria defined for the four dimensions. This established the main parameters for the assignment of categories in the definition of the wild food’s potential. If a wild food does not meet any of these four criteria it may not be relevant for the community (criterion 1.1) or its consumption may have negative effects on the conservation of biodiversity (criterion 2.2) or human health (criteria 3.1 and 3.3). In this case, a Low Potential is assigned indicating that it is not feasible to suggest it as an alternative in the food and nutrition security planning for the territory (Table 2). The foods that through processes of science, technology and innovation demonstrate to comply with the four criteria mentioned above, if they still do not comply with all ten criteria, cannot be suggested as an alternative, but they will have more possibilities of achieving it and therefore could obtain a Moderate or High Potential (Table 2).

A food with Very High Potential will be that which as a result of STI processes obtains the fulfilment of the ten criteria that evaluate the four dimensions proposed by Asprilla-Perea et al. (2020). In this case the food product may be suggested as an alternative in the planning of the nutrition and food security of the territory, since it would not only comply with the criteria of maximum relevance, but with all the criteria that according to these authors evaluate the four dimensions that define the potential of a wild food (Table 2).

The method proposed does not intend to present itself as a strict and inflexible method regarding the evaluation of the potential of wild foods in the nutrition and food security of tropical forests. On the contrary, the present study seeks to encourage discussions on methods for the multidisciplinary estimation of wild foods potential. This type of food constitutes a daily reality in different tropical territories of the planet (Robinson and Bennett 2000; Townsend and Rumiz 2003; Binu 2010; Asprilla-Perea and Hinestroza 2011; Narayanan et al. 2011; Nasi et al. 2011; Pauro et al. 2011; Asprilla-Perea et al. 2012; Keatinge 2012; Martínez-Pérez et al. 2012; Chandra et al. 2013; Kamga et al. 2013; Misra et al. 2013; Álvarez-Salas 2014; Cruz et al. 2014; Grados and Peláez 2014; Saha et al. 2014; Bortolotto et al. 2015; Fa et al. 2015; FAO 2019; Mozhui et al. 2020) and, despite this, they do not officially integrate the planning of food security policies.

In this sense, the method designed and the discussion we hope to promote by it, also offer the academic community a tool to strengthen the process of understanding human/wildlife relationships. This is especially important when certain illnesses of wild animal origin spread rapidly and globally and require planning measures to control such developments. Experimenting with this method in the town of Tutunendo in Northwestern Colombia not only allowed adjustment of the theoretical version of the method to a viable version in its application, but also allowed recognition of the potential of two wild foods used in the area.

In the case of *Alibertia patinoi* (Borojó), a High Potential was obtained that demonstrates the importance of the fruit for this human community; the existence of adequate mechanisms for sustainable use in the territory; a similar nutritional value (at least some nutritional characteristics) to that of other non-wild fruits; and that intake does not generate risks to human health.

In the case of *Proechimys semispinosus*, although advances in STI are evident, its result was a Low potential, mainly due to the lack of a proven sustainable use model in the territory. In neither case was Very High Potential achieved. That means that the fulfilment of some minimum criteria still must be considered viable when planning nutrition and food security in this territory.

It is known that some of the evaluation criteria suggested in this study based on Asprilla-Perea et al. (2020) are not common in its realization, especially in rural territories. However, it is considered that this is not a limitation for the application of the method, since it is based on the recognition of rigorous advances in science and technology existing on the food studied. Moreover, the use of these criteria allows the realization of primary studies to generate non-existent information or with little rigorous treated results.

During the process of formulating the proposed method and its experimental application, our approach has not prioritized the generation of an easy and quick proposal in its application to evaluate wild foods, but a holistic methodology that integrates the different elements that have been identified as structural for the assessment of their potential. Likewise, it is not intended to suggest the use of wild foods where they are not consumed, but rather to provide a tool so that from the academic and scientific field contributions are made to the responsible consumption of this type of food. The results obtained from the application of the method proposed have the potential to generate alerts that discourage the use or frequency of use of any food whose consumption may generate problems for human health or the conservation of biodiversity. This aspect could have a high impact on the recognition of possible traditional and cultural practices with a high risk of transmission of pathogens due to the inappropriate handling and/ or consumption of wild foods. This early recognition will contribute to the prevention of diseases, including those that are rapidly spreading worldwide.

Finally, policies, programmes and projects in favour of food security that include wild foods with Very High Potential could be more realistic and significant, in terms of sustainability and relevance, than those that do not include them, especially in territories where this type of food is used by tradition. Of course, this hypothesis must be tested in subsequent studies. In this line, the inclusion of wild foods in the planning of the nutrition and food security of territories associated with tropical forests has been limited by various challenges that are related to their sustainable consumption. In this regard, future perspectives of this research should address a monitoring process of the interventions in favour of nutrition and food security that include wild foods with Very High Potential in their planning. The objective would be to confirm or discard the contributions of these interventions to nutrition and food security, human health and the conservation of the environment; as well as their relevance and sustainability, and their effects on the food sovereignty of the territory.
